# Operationalizing Quality Measurement in Long-Term Care: A Policy Review and Phased Implementation Framework for Greece

**DOI:** 10.3390/healthcare14131951

**Published:** 2026-07-01

**Authors:** Maria Gamvrouli, Christos Triantafyllou, Joao Breda

**Affiliations:** World Health Organization (WHO) Athens Quality of Care and Patient Safety Office, 10675 Athens, Greece; mairitaga@hotmail.com (M.G.); rodriguesdasilvabred@who.int (J.B.)

**Keywords:** long-term care, quality indicators, healthcare operations management, Greece, patient safety, provider dashboards, performance measurement, ageing, health system governance, quality improvement

## Abstract

Background/Objectives: Long-term care (LTC) is becoming a strategic priority for health systems facing population ageing, multimorbidity, frailty, and increasing demand for coordinated medical and social support. Greece faces these pressures in a context of fragmented governance, limited formal LTC capacity, heavy reliance on family care, and quality oversight that remains largely compliance-oriented rather than performance-oriented. This policy review aims to translate international and national evidence into an operational framework for measuring and improving LTC quality in Greece. Methods: The review combined a structured search of peer-reviewed literature, international policy reports, statistical sources, and Greek legislative and regulatory texts with a pragmatic feasibility assessment of candidate indicators. Results: Evidence from OECD and EU systems suggests that mature LTC quality systems share four operational features: legally mandated reporting, standardised indicators, public transparency, and use of data for provider-level improvement. For Greece, the analysis identifies major gaps in legal reporting obligations, data interoperability, workforce monitoring, public reporting, and user-experience measurement. We propose a three-tier indicator framework covering safety, clinical care processes, workforce and staffing, person-centredness, access and equity, efficiency, governance, and digital readiness. Implementation should proceed through a five-year roadmap: foundation, scale-up, and consolidation. Provider-level dashboards and a National LTC Quality Observatory are recommended as key mechanisms for transforming data into continuous quality improvement. Conclusions: A phased, feasible, and legally anchored approach could strengthen patient safety, dignity, operational efficiency, and accountability in Greek LTC.

## 1. Introduction

Long-term care (LTC) includes a broad range of health, personal, and social support services delivered over extended periods to people with reduced functional capacity due to ageing, disability, frailty, dementia, multimorbidity, or chronic illness [1,2,3]. In ageing societies, LTC is no longer a peripheral social service; it is a core component of health system performance, patient safety, fiscal sustainability, and social protection.

Demographic pressure is the most visible driver of LTC demand. The global population aged 65 years and over is projected to rise substantially by 2050, while the share of people aged 80 years and over is expected to increase markedly across Europe [4,5]. These trends are accompanied by a higher prevalence of multimorbidity, frailty, cognitive impairment, and functional limitations, all of which increase the need for integrated, continuous, and person-centred care [6,7].

The operational challenge for policymakers is not only to expand capacity, but to ensure that LTC services are safe, equitable, efficient, responsive, and respectful of users’ dignity and autonomy. Poor quality LTC may contribute to preventable falls, pressure ulcers, infections, avoidable hospital transfers, medication-related harm, loss of independence, and reduced quality of life [8,9,10,11,12]. Conversely, well-designed LTC quality systems can reduce unnecessary acute care use, strengthen workforce planning, and support value-based allocation of scarce resources [2,13,14].

Greece represents a particularly important case. The country is ageing rapidly and has historically relied on family-based and informal care, while formal LTC provision remains limited, fragmented, and unevenly distributed [15,16,17,18]. Governance responsibilities span health, social welfare, and municipal authorities, creating coordination challenges and variable oversight. Existing regulation largely emphasises licensing and compliance with minimum structural standards, rather than routine measurement of outcomes, care processes, user experience, and provider performance [15,18].

International organisations, including the OECD, WHO, and European Commission, have increasingly emphasised the importance of measuring LTC quality through standardised indicators covering safety, workforce capacity, person-centredness, efficiency, and quality of life. Several countries have established national LTC quality reporting systems, provider benchmarking mechanisms, and public transparency initiatives. However, implementation approaches vary substantially across jurisdictions, reflecting differences in governance structures, financing arrangements, digital infrastructure, and workforce capacity.

Unlike existing OECD and European long-term care quality frameworks, which primarily provide conceptual guidance and comparative indicator sets, this paper focuses on the operationalization of quality measurement within the Greek context. Specifically, it proposes a phased three-tier indicator framework linked to implementation feasibility, provider-level performance dashboards, and a national governance mechanism for quality oversight. By integrating international best practices with the organizational, governance, regulatory, and digital realities of the Greek long-term care system, the proposed framework advances current practice by providing an implementation-oriented roadmap for the phased development of quality measurement, accountability, and continuous quality improvement in Greece.

## 2. Materials and Methods

### 2.1. Design

This study is a policy review and framework-development paper. It combines evidence synthesis, policy scanning, and implementation-oriented feasibility assessment. It is not presented as a PRISMA-compliant systematic review because the available project documentation does not include a complete flow diagram, exhaustive screening log, formal risk-of-bias appraisal, or protocol registration. The review was designed to support policy decision-making and operational implementation in the Greek LTC sector.

### 2.2. Review Questions

The review was guided by three policy and operational questions:

Which LTC quality indicators are used in mature OECD and EU systems, and how are they embedded in governance, regulation, public reporting, and provider improvement?

What are the main gaps in LTC quality monitoring, governance, and data infrastructure in Greece?

Which indicators are immediately feasible, which require short- to medium-term development, and which should be considered aspirational for Greece?

### 2.3. Sources and Search Strategy

The review drew on four evidence streams: peer-reviewed literature, international policy documents, Greek legislation and regulatory texts, and statistical or administrative data sources. Peer-reviewed literature was searched primarily through PubMed and Scopus. Representative search terms included combinations of: (“long-term care” OR “nursing home” OR “home care”) AND (“quality indicators” OR “quality measurement” OR “performance measurement” OR “quality monitoring”) AND (“governance” OR “public reporting” OR “provider dashboards” OR “quality improvement”). Search terms were adapted according to database requirements and supplemented by targeted searches of policy documents and legislative sources. The search focused primarily on literature published between 2000 and 2025, reflecting contemporary developments in long-term care quality measurement and governance. Seminal publications and foundational quality frameworks, including Donabedian’s structure–process–outcome model, were included irrespective of publication date because of their continuing conceptual relevance.

Policy sources included OECD, World Health Organization (WHO), European Commission, World Bank, and national LTC quality frameworks from selected OECD and European countries. Greek legislative texts, presidential decrees, ministerial provisions, and relevant policy documents were reviewed to identify the current legal basis for LTC governance and quality oversight.

Searches were conducted in English and Greek to capture both international evidence and domestic policy material. Foundational quality frameworks, including Donabedian’s structure–process–outcome model, were included despite older publication dates because of their continuing conceptual relevance [19,20].

Overall, the final evidence base comprised 28 included documents. These included peer-reviewed journal articles, OECD and WHO reports, European Commission publications, national long-term care quality frameworks, and Greek legislative and policy documents relevant to LTC quality measurement and governance. Sources were prioritised according to their operational relevance, implementation experience, and applicability to the Greek LTC system.

### 2.4. Eligibility Criteria

Sources were eligible if they addressed LTC quality, quality indicators, monitoring frameworks, public reporting, provider performance, workforce and staffing, person-centredness, safety, data systems, or governance mechanisms relevant to LTC. Sources were excluded if they focused exclusively on acute hospital care, presented opinion without operational or empirical content, or lacked relevance to system-level or provider-level quality measurement.

For peer-reviewed studies, eligibility focused on LTC quality measurement frameworks, indicator development, implementation experiences, and governance mechanisms. For policy reports and international guidance documents, eligibility was based on relevance to LTC quality governance, reporting systems, and performance measurement. For legislative and regulatory sources, eligibility depended on their relevance to LTC governance, oversight, quality assurance, or provider accountability.

### 2.5. Data Extraction and Synthesis

For each source, information was extracted on jurisdiction, quality domains, indicator definitions, data sources, reporting arrangements, governance structures, implementation mechanisms, and barriers or enablers. The synthesis was narrative and implementation-oriented rather than statistical. Sources were prioritised according to their relevance to LTC quality measurement, operational applicability, transferability to the Greek context, and usefulness for policy and provider-level implementation. Particular attention was given to sources that described standardised indicators, legally mandated reporting, provider dashboards, public reporting systems, workforce measures, user-experience measures, and governance mechanisms.

The extracted evidence was synthesised narratively and mapped against priority domains for LTC quality: safety, clinical care processes, workforce and staffing, person-centredness, access and equity, efficiency and sustainability, governance and quality improvement, and digital readiness. Evidence was then interpreted in relation to the Greek LTC context, including current governance fragmentation, limited formal LTC capacity, reliance on informal care, variable provider capacity, and emerging digital health infrastructure.

### 2.6. Feasibility Assessment

Candidate indicators were classified into three tiers according to their suitability for phased implementation in Greece. The feasibility assessment considered data availability, technical complexity, legal feasibility, provider burden, alignment with national policy priorities, and potential usefulness for management and regulatory decision-making.

The assessment was conducted primarily as an author-driven policy and implementation analysis informed by the authors’ expertise in healthcare quality measurement, health system governance, and long-term care policy. The analysis also benefited from ongoing discussions with policymakers, technical experts, and stakeholders involved in quality-of-care initiatives in Greece. However, no formal Delphi process, structured stakeholder consultation, expert consensus exercise, or pilot feasibility testing formed part of the study methodology.

Tier 1 indicators were considered immediately feasible and suitable for mandatory national reporting. Tier 2 indicators were considered feasible in the short to medium term but required additional training, IT capacity, standardisation, or piloting. Tier 3 indicators were considered aspirational and dependent on integrated digital infrastructure, advanced analytics, or mature outcome-measurement systems.

## 3. Results

### 3.1. Conceptual Basis for LTC Quality Measurement

The Donabedian framework remains highly relevant for LTC quality measurement because it links structural capacity, care processes, and outcomes [19,20]. In LTC, structure includes staffing, infrastructure, financing, governance, digital systems, and provider capacity. Process indicators capture what providers do, including care planning, medication review, falls prevention, nutrition screening, pain assessment, continence care, advance care planning, user engagement, and incident reporting. Outcome indicators capture the effects of care, including falls with injury, pressure ulcers, hospital transfers, functional decline, quality of life, satisfaction, dignity, and participation.

However, LTC quality cannot be understood only through clinical safety indicators. Contemporary frameworks increasingly emphasise rights, autonomy, social participation, dignity, choice, family involvement, equity, and the lived experience of users and carers [21,22,23,24,25]. This requires combining objective operational indicators with user-reported experience measures and carer feedback.

Integration across health and social care is another essential quality dimension. Many poor LTC outcomes arise at interfaces: hospital discharge to residential care, transitions between home care and primary care, medication reconciliation, emergency transfers, and information exchange between providers [11,12,26]. Therefore, an LTC quality framework should not only measure what happens inside individual facilities, but also how well providers coordinate with the broader health system.

### 3.2. International Lessons for LTC Quality Systems

First, quality reporting is legally or contractually mandated. Countries with stronger LTC quality systems embed quality measurement into regulation, licensing, payment systems, or national care standards. This creates stable expectations for providers and enables consistent data collection over time [2,27,28,29,30,31].

Second, indicator sets are standardised and nationally comparable. Common domains include safety, workforce, clinical care processes, user experience, access, equity, and efficiency [2,27,28,32,33,34]. The most frequently used indicators include falls, pressure ulcers, medication errors, unplanned hospital transfers, vaccination coverage, staffing levels, staff turnover, care planning, user satisfaction, and complaints management.

Third, public reporting is used to increase transparency and accountability. Public reporting systems, such as facility-level ratings, inspection reports, or quality portals, can support informed user choice, encourage providers to improve, and help regulators target oversight [35,36,37]. The effect of public reporting is generally stronger when paired with quality improvement support rather than used only as a punitive tool.

Fourth, data are used for operational management, not only for external control. Provider-level dashboards, benchmarking tools, and integrated data systems allow managers to identify risks, track trends, compare performance, and prioritise improvement activities. Examples from Canada, Finland, Scotland, and other systems demonstrate the value of linking LTC data with health and social care datasets [33,34,38,39].

### 3.3. The Greek LTC Context

Greece’s LTC system is shaped by demographic ageing, limited formal provision, strong dependence on family and informal carers, and fragmented institutional responsibilities [15,16,17,18]. Formal LTC services include residential care facilities, municipal home care programmes, day centres, and non-profit or private providers. However, availability varies geographically, and rural and island areas may face greater access constraints.

Governance is divided across the Ministry of Health, the Ministry of Labour and Social Affairs, municipalities, social welfare structures, and health insurance or administrative bodies. This fragmentation creates challenges for quality governance, data standardisation, workforce planning, and integrated service delivery [15,18,40].

Current oversight focuses mainly on licensing, infrastructure, staffing requirements, and compliance with minimum standards. Greece does not yet have a national, routine, standardised LTC quality indicator system; a public reporting portal for LTC provider performance; a national LTC quality observatory; or systematic integration between LTC quality data and health system datasets.

Nevertheless, there are important policy opportunities. Existing Greek legislation provides a foundation for regulation, patient rights, social care governance, and quality assurance. In addition, digital health reforms, European policy momentum, and the broader quality-of-care agenda create favourable conditions for introducing structured LTC quality measurement [41].

### 3.4. Main Gaps in Greece

The analysis identified six major gaps.

First, there is no legal mandate requiring all LTC providers to submit standardised quality indicators. Without such a mandate, routine measurement remains optional, fragmented, and difficult to compare.

Second, available data systems are not designed for routine LTC quality measurement and monitoring. Administrative, claims, inspection, vaccination, and provider data exist but are not integrated into a coherent performance framework.

Third, user experience and dignity are under-measured. There is limited routine measurement of satisfaction, autonomy, respect, choice, quality of life, family experience, and complaint resolution.

Fourth, workforce indicators are insufficiently developed. Staffing ratios, staff turnover, training compliance, skill mix, absenteeism, and workforce stability are central to quality, but they are not consistently measured or publicly reported.

Fifth, provider-level dashboards are not routinely available. Managers and regulators, therefore, lack real-time or near-real-time tools for identifying quality risks and monitoring improvement.

Sixth, public transparency remains limited. Inspection findings and provider performance metrics are generally not accessible in a way that supports accountability, informed choice, and public trust.

### 3.5. Proposed Three-Tier LTC Quality Measurement and Implementation Framework for Greece

A phased framework is proposed to balance ambition with feasibility. The framework should be interpreted as a policy implementation model designed to guide the progressive development of LTC quality measurement in Greece rather than as a validated national indicator set. The framework includes eight domains: safety and risk management; clinical care processes; workforce and staffing; person-centredness and user experience; access and equity; efficiency and sustainability; governance and quality improvement; and innovation and digital readiness (Table 1).

Tier 1 indicators should be prioritised for immediate national reporting because they are practical, understandable, and relevant to patient safety, operational management, and public accountability. Tier 2 indicators should be piloted and gradually scaled as provider capacity grows. Tier 3 indicators should be treated as long-term goals requiring interoperable digital systems, advanced analytics, and mature quality governance.

Tier 1 indicators were prioritised because they combine high policy relevance with relative operational feasibility. These indicators address core risks for LTC users, including falls, infection outbreaks, vaccination coverage, unplanned hospital transfers, staffing capacity, user satisfaction, complaints resolution, and incident reporting. They are also more likely to be collectable through basic provider reporting, existing administrative data, inspection mechanisms, or simple dashboard systems. In contrast, Tier 2 and Tier 3 indicators require greater standardisation, stronger digital infrastructure, more advanced outcome measurement, risk adjustment, or longitudinal data linkage.

The prioritisation of Tier 1 indicators should therefore be understood as a pragmatic starting point rather than a final national indicator set. Before national rollout, these indicators should be tested through pilot implementation across different LTC settings, including residential facilities, municipal home care programmes, and community-based providers. This would allow Greece to refine definitions, assess reporting burden, evaluate data quality, and ensure that indicators are useful for both providers and regulators.

**Figure 1:**
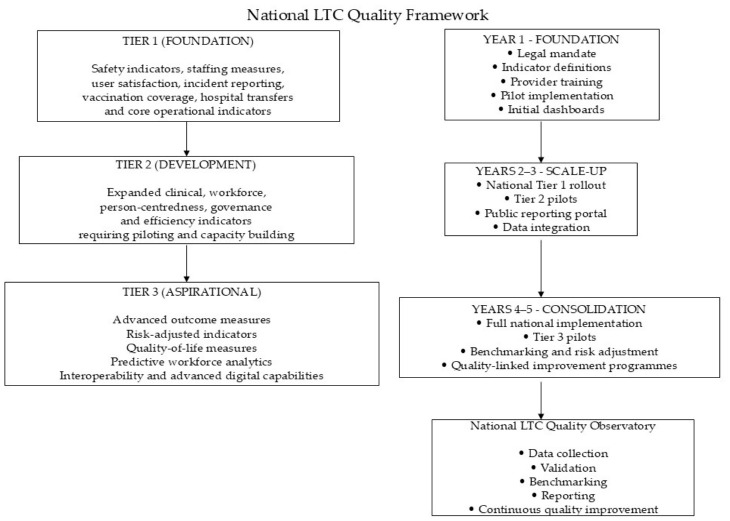
Proposed Three-Tier Long-Term Care Quality Measurement Framework and Implementation Roadmap for Greece.

The proposed framework is summarised in Figure 1, which illustrates the relationship between the three implementation tiers, the phased rollout strategy, and the governance mechanisms required to support sustainable quality measurement and improvement across the Greek LTC sector.

### 3.6. Implementation Roadmap

A five-year phased implementation roadmap is proposed (Table 2).

Phase 1: Foundation, Year 1

The first phase should establish the legal, technical, and organisational foundations. This includes legislative reform to require Tier 1 quality indicator reporting as part of licensing or contractual obligations; creation of a National LTC Quality Observatory or equivalent inter-ministerial function; development of standard indicator definitions; preparation of reporting templates; provider training; and pilots across a representative sample of residential, home care, and community-based LTC providers.

Phase 2: Scale-up, Years 2–3

The second phase should expand Tier 1 reporting nationwide and begin piloting Tier 2 indicators. Data collection should be integrated with existing administrative and health information systems wherever possible, including claims, vaccination, inspection, and provider reporting systems. A public reporting portal should be launched initially with aggregated national and regional results, followed by provider-level reporting once data quality is sufficiently robust.

Phase 3: Consolidation and Expansion, Years 4–5

The third phase should consolidate national reporting, introduce selected Tier 3 indicators in advanced providers, establish benchmarking and risk adjustment, and link quality results to improvement support, training, inspection planning, and eventually financial incentives. Greece should also aim to align its framework with OECD and European LTC quality measurement initiatives.

### 3.7. Provider-Level Dashboards

Provider-level dashboards are essential for converting measurement into management action. A dashboard should present clear, timely, and actionable data to facility managers, quality teams, inspectors, policymakers, and eventually the public.

Core dashboard functions should include trend monitoring, benchmarking against regional or national averages, alerts for deteriorating indicators, and drill-down capacity by unit, shift, population group, or care process. Visual formats such as charts, traffic-light coding, and performance summaries can make data usable for non-technical decision-makers.

A minimum LTC dashboard for Greece could include staff-to-resident ratios, falls with injury, vaccination coverage, unplanned hospital transfers, user satisfaction, complaints resolution, incident reporting, staff training compliance, and infection outbreaks. More advanced dashboards could include pressure ulcers, ADL decline, pain management, avoidable hospital admissions, social participation, and quality-of-life indicators.

Provider dashboards should be used for three purposes: internal quality improvement, regulatory oversight, and public transparency. Internally, they can help managers identify early warning signs and design improvement interventions. For regulators, they can support risk-based inspections. For the public, they can provide accessible and comparable information once data quality and risk adjustment are sufficiently mature.

Effective dashboard implementation will require clear data governance arrangements, standardised indicator definitions, routine validation procedures, and mechanisms for ensuring data completeness and accuracy. Before provider-level public reporting is introduced, appropriate risk-adjustment methodologies should be developed to minimise unfair comparisons and account for differences in resident complexity and provider case mix.

## 4. Discussion

This policy review highlights a central challenge for Greece: LTC quality cannot be improved at scale unless it is measured, governed, and made actionable. The country faces rapidly rising demand for LTC, yet its quality oversight remains largely compliance-based and fragmented. This creates blind spots for policymakers, providers, users, and families.

The proposed framework responds to this challenge by offering a pragmatic policy implementation model that bridges international best practice and national feasibility. It is intended to support the phased development of LTC quality measurement in Greece and should not be interpreted as an empirically validated indicator system. Rather than recommending immediate adoption of a large and complex indicator set, it proposes a staged approach that begins with a small number of high-value, feasible indicators. This is consistent with international experience showing that quality systems are more likely to succeed when they begin with clear definitions, manageable reporting requirements, and practical use of data [2,27,28].

Legal anchoring is critical. Voluntary reporting alone is unlikely to produce complete, comparable, or sustainable data. A legal requirement for routine quality reporting should therefore be embedded in licensing, provider contracts, or regulatory obligations. However, legal reform should be accompanied by training, technical assistance, and clear communication that the purpose of measurement is improvement as well as accountability.

The creation of a National LTC Quality Observatory would help overcome fragmentation by centralising indicator definitions, data collection, validation, analysis, reporting, and technical support. Such an observatory could be housed within an existing institution or established as a joint inter-ministerial mechanism involving health, social care, municipalities, providers, professional bodies, users, and carers.

The proposed provider dashboards are particularly relevant to the Special Issue theme of optimizing healthcare operations and management. Dashboards can transform LTC quality from an abstract policy concept into a daily management tool. They can help managers allocate staff, identify safety risks, reduce preventable hospital transfers, monitor user satisfaction, and target training where it is most needed. In this sense, the dashboard is not merely a reporting instrument; it is an operational management infrastructure [33,34,38,39].

Workforce measurement deserves special attention. International evidence consistently links staffing levels, skill mix, training, and turnover with quality of care in nursing homes and other LTC settings [9,10,14,32]. In Greece, where LTC workforce capacity and recognition remain major challenges, workforce indicators should be treated as a core quality domain rather than as administrative statistics.

Person-centredness is equally important. LTC quality should not be reduced to clinical safety alone. For older people and people with disabilities, quality also means dignity, autonomy, meaningful activity, social connection, family involvement, and control over daily life [21,22,23,24,25]. Therefore, user and family experience measures should be embedded in the national framework from the beginning.

Implementation in Greece will require careful attention to administrative, financial, and workforce-related barriers. Governance fragmentation remains a major challenge, as LTC responsibilities are distributed across health, social welfare, municipal authorities, private providers, and non-profit organisations. Without a clearly designated coordinating body, quality measurement may remain fragmented and inconsistently applied. For this reason, the proposed National LTC Quality Observatory should not only collect data but also define indicators, validate submissions, coordinate reporting standards, support providers, and facilitate inter-ministerial collaboration.

Financing is another critical condition for implementation. Mandatory reporting without dedicated resources could increase administrative burden and generate resistance among providers. A feasible approach would therefore require phased financing for provider training, digital reporting tools, data validation, and technical support. Where possible, Greece should build on existing administrative, inspection, vaccination, and provider-reporting systems rather than creating parallel reporting structures.

Workforce shortages and skill-mix limitations may also affect implementation. LTC providers may have limited capacity to collect, interpret, and use quality data, particularly in smaller facilities or municipal home care services. Therefore, quality measurement should be accompanied by practical training, simplified indicator manuals, feedback reports, and gradual implementation. The initial focus should be on a limited number of Tier 1 indicators that are meaningful, feasible, and directly linked to safety and service improvement.

Finally, implementation should avoid being perceived as a punitive inspection exercise. Quality reporting should be linked to learning, benchmarking, technical assistance, and gradual public transparency. Provider-level reporting should only be introduced once indicator definitions, data quality, and risk-adjustment procedures are sufficiently mature. This phased approach would increase trust, reduce resistance, and improve the likelihood that quality measurement becomes a tool for continuous improvement rather than administrative compliance alone.

The risks of implementation are real. Poor data quality, excessive reporting burden, provider resistance, underdeveloped IT systems, and insufficient staff capacity could undermine the initiative. These risks can be mitigated through phased implementation, simple Tier 1 indicators, piloting, training, feedback loops, and careful communication. Risk adjustment will also be needed over time to ensure that providers serving more complex or disadvantaged populations are not unfairly penalised.

The proposed approach has limitations. It is based on a policy review and feasibility assessment rather than a full systematic review with formal risk-of-bias appraisal. Although the methods were strengthened to describe the search process, source selection, and synthesis procedures, the review was designed to support policy development and implementation planning rather than evidence synthesis according to systematic review standards. Furthermore, although the feasibility assessment was informed by the authors’ expertise and by ongoing discussions with policymakers and technical experts involved in quality-of-care initiatives in Greece, it did not include a formal Delphi process, structured stakeholder consultation, expert consensus methodology, or pilot feasibility testing. Some Greek data sources remain unpublished or fragmented, and the feasibility of specific indicators may evolve as digital infrastructure, financing mechanisms, and LTC governance arrangements mature. Future work should therefore include formal stakeholder validation, pilot testing of Tier 1 indicators, and empirical assessment of reporting burden, data quality, and provider-level usefulness. Accordingly, the framework should be considered an implementation-oriented policy proposal whose indicators and governance arrangements require empirical validation before national adoption.

## 5. Conclusions

This paper proposes an implementation-oriented policy model to support Greece’s transition from minimum compliance in long-term care oversight toward a more structured quality measurement and improvement system. International experience shows that effective LTC quality systems require legal mandates, standardised indicators, public transparency, provider-level dashboards, and governance structures capable of turning data into action.

A phased three-tier indicator framework may provide Greece with a feasible pathway for strengthening LTC quality measurement while recognising current limitations in governance, workforce capacity, data infrastructure, and reporting systems. Tier 1 indicators may be suitable for initial piloting and progressive implementation, whereas Tier 2 and Tier 3 indicators will require additional validation, infrastructure development, and implementation readiness.

Before large-scale implementation, further work should include stakeholder consultation, pilot testing across different LTC settings, refinement of indicator definitions, assessment of reporting burden, evaluation of data quality, development of risk-adjustment methodologies, and strengthening of digital infrastructure. Such efforts will help ensure that LTC quality measurement supports continuous learning, transparency, accountability, and person-centred care.

The central policy message is clear: LTC quality measurement should not be treated as an administrative burden, but as an essential infrastructure for safer, more dignified, more efficient, and more person-centred care.

## Figures and Tables

**Table 1:** Proposed three-tier LTC quality indicator framework for Greece.

Domain	Tier 1 (Immediate)	Indicative Data Source	Reporting Frequency	LTC Setting
Safety & Risk Management	Falls with injury; unplanned hospital transfers; vaccination coverage; infection outbreaks	Provider reports; hospital data; vaccination registries	Quarterly	Residential, Home Care, Community
Clinical Care Processes	Medication review; nutrition screening; continence care plan	Clinical records	Quarterly	Residential, Home Care
Workforce & Staffing	RN hours/resident-day; care worker hours; training compliance	HR systems	Quarterly	All settings
Person-Centredness	User satisfaction; respect and dignity; complaints resolved	User surveys; complaint systems	Biannually	All settings
Access & Equity	Waiting time; home care coverage	Administrative systems	Annually	All settings
Efficiency & Sustainability	Bed occupancy; length of stay	Provider administrative data	Quarterly	Residential
Governance & Quality Improvement	Quality improvement plan; internal audits; incident reporting compliance	Quality reports	Annually	All settings
Innovation & Digital Readiness	Basic EHR use; digital skills training	Provider self-assessment	Annually	All settings

Note: Detailed technical specifications, including indicator definitions, numerators, denominators, exclusions, risk-adjustment requirements, and validation procedures, should be developed during pilot implementation and incorporated into national LTC reporting guidance.

**Table 2:** Implementation roadmap for a national LTC quality measurement.

Phase	Responsible Actors	Main Actions	Dependencies	Risks	Expected Outputs
Foundation (Year 1)	Ministry of Health; Ministry of Labour and Social Affairs; Municipalities; National LTC Quality Observatory	Legal framework; Tier 1 indicator definitions; pilot implementation; provider training	Legislative support; stakeholder engagement	Resistance to reporting; limited provider capacity	Indicator manual; pilot dashboard; reporting templates
Scale-Up (Years 2–3)	Ministries; Observatory; Providers; Professional Bodies	National Tier 1 rollout; Tier 2 pilots; data integration; public reporting portal	Data quality; IT infrastructure	Reporting burden; inconsistent data submission	National LTC dataset; regional benchmarks
Consolidation (Years 4–5)	Observatory; Regulators; Providers	Tier 3 pilots; benchmarking; risk adjustment; quality-linked incentives	Mature data systems; workforce capacity	Data gaming; inequitable comparisons	National benchmarking system; provider dashboards; EU/OECD alignment

## Data Availability

This study did not generate any new data.

## Abbreviations

The following abbreviations are used in this manuscript:

ADL	Activities of Daily Living
CIHI	Canadian Institute for Health Information
EHR	Electronic Health Record
EU	European Union
LTC	Long-Term Care
OECD	Organisation for Economic Co-operation and Development
WHO	World Health Organization
